# Burkitt lymphoma presenting as cecum mass and spontaneous tumor lysis syndrome with hypercalcemia

**DOI:** 10.1002/ccr3.6977

**Published:** 2023-02-23

**Authors:** Mahan Shafie, Elnaz Shahmohamadi, Hoomaan Ghasemi, Narjes Zarei Jalalabadi, Samaneh Parsa

**Affiliations:** ^1^ School of Medicine Tehran University of Medical Sciences Tehran Iran; ^2^ Department of Internal Medicine, Imam Khomeini Hospital Complex Tehran University of Medical Sciences Tehran Iran

**Keywords:** Burkitt's lymphoma, cecum cancer, hypercalcemia, spontaneous tumor lysis syndrome

## Abstract

Burkitt's lymphoma (BL) could be primarily presented with various symptoms. We reported a woman with abdominal pain and mass who later developed spontaneous TLS with hypercalcemia, and was diagnosed with BL. Clinicians should suspect BL in case of any abdominal mass, especially with an aggressive course, to avoid further complications.

## Abstract 2

Immediate and intensive action and treatment are required in patients with life‐threatening Burkitt lymphoma and tumor lysis syndrome. So, a high degree of clinical suspicion is needed to make the correct diagnosis and proceed with treatment.

## 1 INTRODUCTION

Burkitt's lymphoma (BL) is a severe non‐Hodgkin's lymphoma that is typically diagnosed in children and adolescents, and to a lesser extent, middle‐aged people and immunodeficient, endemic, and sporadic (non‐endemic) variations of the disease have been identified.^1^ The presentation and clinical symptoms of BL could vary depending on the form of BL and the primary involved organ, which could mimic other differential diagnoses.^2^ Tumor lysis syndrome (TLS) is an oncologic emergency that may occur spontaneously in a patient with BL, as cells undergo lysis and release their content into circulation. Therefore, metabolic disturbances occur which usually result in hyperphosphatemia, hypocalcemia, hyperkalemia, and hyperuricemia.^3^ Herein, we report a case of a 32‐year‐old female presenting with 3 weeks of abdominal pain and a cecum mass who developed spontaneous TLS during hospitalization, and due to laboratory findings, she was eventually diagnosed with Burkitt lymphoma.

## 2 CASE PRESENTATION

**FIGURE 1:**
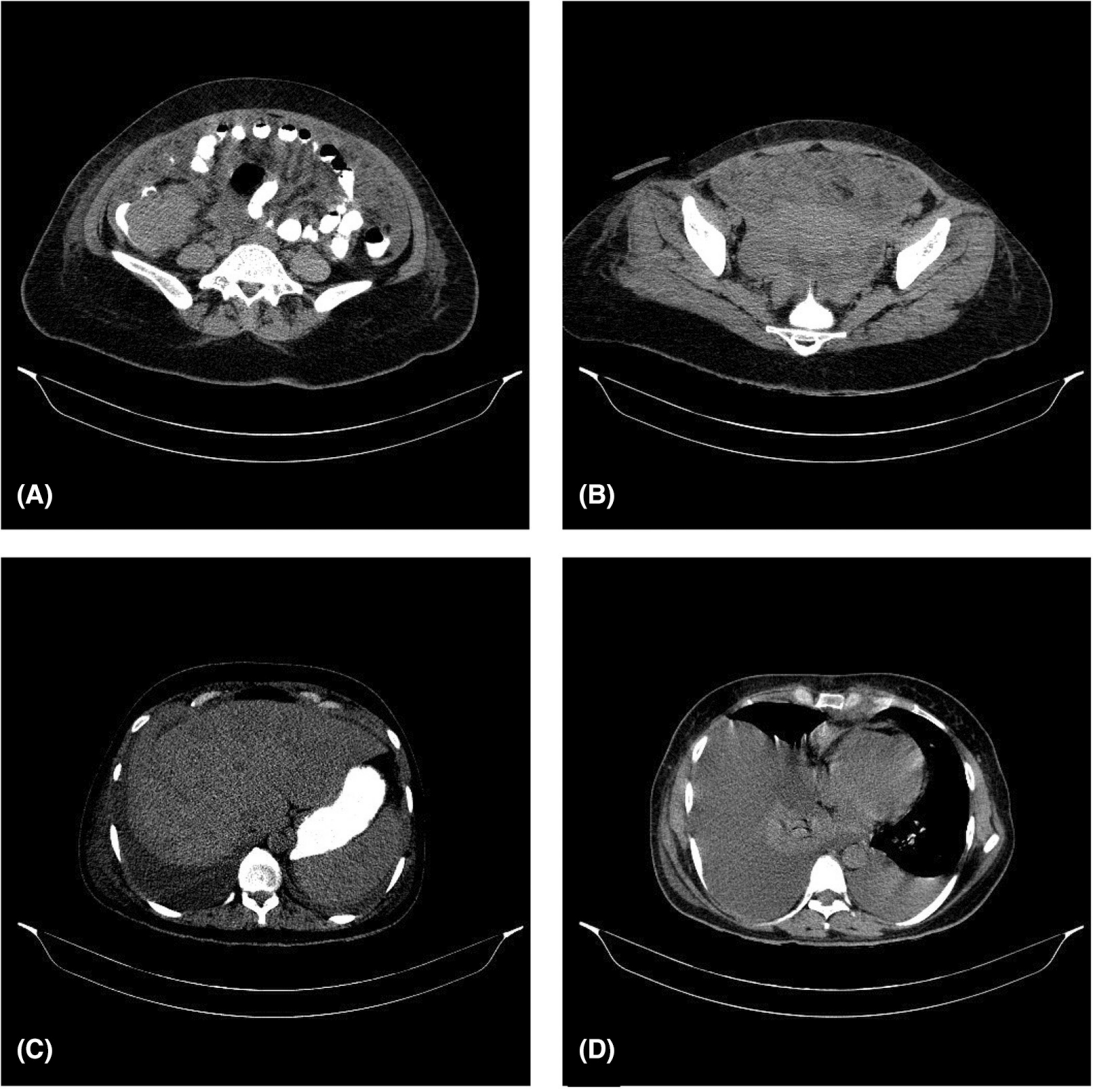
Thoracoabdominal CT scan with oral contrast showing: (A) cecal mass, (B) omental infiltration, (C) liver lesions, and (D) pleural effusion.

A 32‐year‐old Persian female presented with abdominal pain for 3 weeks and had been undergoing several workups before her visit. She was previously subjected to a colonoscopy which identified a cecum mass with a presumptive diagnosis of colon cancer. However, she was referred to our hospital with ascites, dyspnea, weakness, and malaise. The patient did not have any significant past medical history, did not take any medication, and had no family history of malignancy. She was noted to be tachycardic, tachypneic, and hypoxic and admitted for consideration of metastasis and to do further workups. On examination, the breath sounds were decreased in both lower lung fields and no rales were heard. Venous blood gas analysis revealed a pH value of 7.31 (normal range 7.35–7.45); PCO2 of 21.6 (normal range 35–45); and PO2 of 98 (normal range 80–100). Complete blood count results were as follows: WBC: 7100/μL; Hb: 10.8 g/dL; platelet: 91,000/mm^3^. On admission, laboratory findings indicated creatinine level of 5.6 mg/dL (normal range of 0.6–1.5 mg/dL), potassium level of 5.6 mg/dL (Normal range 3–5.4 mg/dL), calcium level of 10.9 (Normal range 8.6–10.3), and uric acid level was 18.1 mg/dL (Normal range 3.5–7.5 mg/dL). Other significant laboratory values are summarized in Table 1. The patient underwent computed tomography (CT) of the chest and abdomen which revealed a cecal mass, omental infiltration, hypodense lesions in the right lobe of the liver, and pleural effusion (Figure 1). Moreover, due to dyspnea and diminished breath sound, a CT scan of the chest was performed which showed bilateral pleural effusion. She underwent thoracocentesis and a total of 400 cc of sanguineous fluid was drained and a pleural catheter was placed. The pleural fluid contained 11,200 WBCs with the differential showing 80% polys, 20% lymphocytes, and 15,930 U/L lactate dehydrogenase (LDH) with simultaneous serum LDH of 9900 U/L. Given that the patients had findings of electrolyte abnormalities including hyperkalemia, hyperphosphatemia, and hyperuricemia which met the Cairo and Bishop criteria for TLS, suspicion was high for the possibility of spontaneous TLS as the cause of the patient's acute kidney injury. However, due to high levels of LDH in pleural fluid and hypercalcemia, despite what is expected in TLS in solid tumors, we doubted that this is a case of hematologic malignancy, regarding the fact that Burkitt's lymphoma can appear as a mass, particularly in the cecum. Hematology‐oncology was consulted and recommendations were given including bone marrow aspiration and biopsy. Bone marrow section showed monomorphic small mature lymphocytes with high proliferative activity in crushed marrow spaces with extensive necrosis. Significant decrease in trilineage population of hematopoietic elements and megakaryocytes were also observed. Immunohistochemistry (IHC) study resulted in negative CD3, strong positive CD20, negative CD34, negative TdT, positive CD79a, Positive CD10, negative BCL2, and inconclusive ki67, all suggestive for BL.

**TABLE 1:** Laboratory findings of the patient during hospitalization.

	On admission	Day 1	Day 2	Day 3	Day 4	Day 5	Day 6	Day 7	Day 8	Day 9
WBC (×1000/mm^3^)	7.1	6		3.3	4.9	4.8	9.1	10.2	10.6	12.2
RBC (million/mm^3^)	3.96	3.55		2.8	3.02	3.1	3.11	2.94	3.52	3.49
Hg (g/dL)	10.8	9.7	9.2	7.5	8.2	8.1	8.7	7.5	9.6	9.7
MCV (fl)	81.8	82.5		81.4	82.5	82.1	80.7	81.3	83.5	82.1
PLT (×1000/mm^3^)	91	91	69	45	34	28	21	18	14	18
Uric acid (mg/dL)	18.1	18.1	18.2		1.4	1.6		2	2.6	
Sodium (mg/dL)	143	143	145	147	147	136	140	138	141	135
Potassium (mg/dL)	6.2	6.4	6.8	4.2	4.4	4	4.2	4.2	4.4	4.9
Calcium (mg/dL)	10.9	10.3	10.9	8.6	8.1		7.9	7.6	8.8	10.7
Phosphorus (mg/dL)		3.6	5.6	5.7	4.2		3.0	3.4	3.1	2.8
Magnesium (mg/dL)	1.7	1.5	2.3	1.7	1.5		1.5	1.5		1.8
Creatinine (mg/dL)	5.6	4.9	5.1	3.4	2.8		2	1.8	1.4	1.9
BUN (mg/dL)	83	84	113	95	68			61		69
Total protein (g/L)		5.3	5							
LDH (IU/L)	316		9900							9998
BS (mg/dL)	70	92	74	131	131			90	105	67
AST (IU/L)	94	86								
ALT (IU/L)	55	46								
ALP (IU/L)	1434	1035								
GGT (IU/L)		341								
Bili Rubin total (mg/dL)	0.7	2								
Bili Rubin direct (mg/dL)	0.3	0.5								
Plural fluid										
LDH (IU/L)			15,930							
Albumin (g/L)			2000							
WBC (×10^9^/L)			11,200							
RBC (×10^9^/L)			70							
Blood culture			Neg		Neg					

Abbreviations: ALP, alkaline phosphatase; ALT, alanine aminotransferase; AST, aspartate aminotransferase; BS, blood sugar; BUN, blood urea nitrogen; GGT, gamma glutamyl transferase; Hg, hemoglobin; LDH, lactate dehydrogenase; MCV, mean corpuscular volume; PLT, platelet; RBC, red blood cell; WBC, white blood cell.

As TLS is a potentially life‐threatening condition, particularly due to underlying hematologic malignancy, immediately after the diagnosis, hydration initiated for the patient with crystalloid fluids and received treatment with a hypouricemic agent and furosemide. Due to a lack of improvement in her renal function and laboratory tests, intermittent hemodialysis was initiated and the patient received packed red blood cells, which improved patient's electrolytes panel and condition. However, on the eighth day of hospitalization, the patient's condition deteriorated. She was dyspneic, hypoxic, and anxious. The patient developed severe respiratory distress and became intubated immediately and transferred to the intensive care unit. Unfortunately, on the ninth day of hospitalization, the patient suffered a cardiac arrest, and resuscitation efforts were unsuccessful. After the patient deceased, the results of histopathology and bone marrow aspiration were obtained. Histopathology report from the cecum biopsies showed large immunoblastic cells in favor of BL, consistent with the bone marrow aspiration result.

## 3 DISCUSSION

We presented a young woman with a cecum mass, which was later diagnosed to have BL after the presentation of spontaneous TLS with hypercalcemia during hospitalization. BL is a highly aggressive B cell non‐Hodgkin lymphoma typically presented with a rapidly growing tumor mass with a brief doubling time.^1^ The initial presentation of BL varies depending on the form of BL and primary localization of the disease.^2^ According to the current literature, BL may present with gastrointestinal symptoms such as abdominal pain and mass, which could mimic other differential diagnosis such as gastrointestinal disorders and neoplasms.^4^ A number of studies reported cases of BL presenting as acute appendicitis^5^, ^6^, ^7^ and some studies reported BL presenting with gastrointestinal bleeding.^8^, ^9^

Adult sporadic BL is commonly a disease of young male adults in their twenties and thirties; however, our case was a female in her thirties.^1^ Our patient presented with sporadic BL which was not associated with immunocompromised status (HIV infection) or EBV infection. Therefore, our case reminds the fact that older age, negative history of risk factors, and also unexpected gastrointestinal localization, could not exclude the possibility of BL and should be considered as a differential diagnosis even among these patients.^1^

Our patient initially presented with abdominal pain and accordingly, a cecum mass was identified through a previous colonoscopy. However, during hospitalization, electrolyte abnormalities including hyperkalemia, hyperphosphatemia, and hyperuricemia which met the Cairo and Bishop criteria for TLS,^10^ raised suspicion for probable BL since TLS is a complication observed most frequently in patients with hematologic malignancies such as BL and acute lymphoblastic leukemia (ALL). Moreover, BL may associate with high serum LDH concentration, which was observed in our patient.^11^

Tumor lysis syndrome is an oncologic emergency that is characterized by a set of metabolic abnormalities, including hyperkalemia, hyperphosphatemia, secondary hypocalcemia, and hyperuricemia which are the consequences of massive release of intracellular components (i.e., potassium, phosphate, and nucleic acids) into the systemic circulation following the rapid lysis of malignant cells. Although TLS more commonly presents within 12–72 h after treatment administration, similar to our case, it may coincide without the initiation of cytotoxic therapy.^3^ Our patient presented with hyperkalemia, hyperphosphatemia, hyperuricemia, increased serum creatinine, and sudden death, which met the criteria for laboratory and clinical TLS, but contrary to typical TLS cases, hypercalcemia was detected in initial laboratory findings. Based on the literature, the development of TLS with hypercalcemia is a rare phenomenon reported in a few cases, mainly corresponding to hematological malignancies.^12^, ^13^, ^14^ There are various ways in malignancies including ectopic production of PTH, PTHrP production, production of cytokines, tumor production of vitamin D, and bone metastasis to increase the level of calcium despite the occurrence of TLS.^12^ Of note, hypercalcemia should not delay the diagnosis of TLS since hypercalcemia may occur in patients with malignancy.

Tumor lysis syndrome may occur in patients with both solid tumors and hematologic malignancies. However, patients under treatment for hematologic malignancies, mainly BL and ALL, are at the highest risk for developing TLS.^15^ Based on existing literature, certain intrinsic tumor‐related factors have been associated with an increased risk of developing TLS, such as high tumor cell proliferation rate, significant tumor burden, tumor chemosensitivity, and increased LDH levels, which were all seen in our case. Furthermore, it has been suggested that certain conditions, including preexisting uremia or hyperuricemia, decreased urinary flow or acidic urine, dehydration, oliguria, anuria, and renal insufficiency or renal failure may predispose patients to develop TLS, which was also observed in our case.^15^ A risk stratification system for TLS was proposed based on the type of cancer, the burden of disease, treatment, expected response to treatment, and renal function, which classifies patients into three categories: high risk, intermediate risk, and low risk. The recommended therapy varies according to the risk category.^16^ Our case had a hematologic malignancy (i.e., BL) with extremely high proliferation rate and increased LDH serum level and renal failure; therefore, based on the risk stratification system, our patient is included in the high‐risk group, with a more than five percent risk of TLS.

The development of TLS is associated with increased mortality, and the prognosis is associated with severity of underlying malignancy and the development of renal dysfunction, as was observed in our case.^17^ The potential life‐threatening complications resulting from TLS necessitate preventive care and immediate treatment in events of TLS occurrence especially in cases with BL. The main strategies to prevent TLS are aggressive intravenous (IV) hydration and diuresis, which are recommended in all patients at intermediate or high risk for TLS. The goal of hydration is to maintain a high urine output by improving intravascular volume, renal perfusion, and glomerular filtration to enhance the excretion of uric acid and phosphate. These patients should receive continuous cardiac monitoring, as hyperkalemia may lead to cardiac arrhythmia or sudden death. Hence, calcium gluconate may need to be administered, and potassium‐lowering strategies such as glucose plus insulin, beta‐agonists, and oral potassium‐lowering agents should be considered. Furthermore, hypouricemic agents, including allopurinol and rasburicase, along with phosphate lowering strategies (e.g., hydration and phosphate binder therapy) should be considered to prevent possible acute kidney injury. Despite optimal care, severe acute kidney injury may develop in some patients with TLS as was seen in our case, requiring renal replacement therapy.^18^ In our case, due to the lack of improvement in the patient's renal function and persistent electrolyte abnormalities, hemodialysis was initiated.

To the best of our knowledge, only a few cases of BL initially presented with abdominal pain and a cecum mass. This case also represents a unique educational scenario that reminds physicians that BL may present with gastrointestinal symptoms such as an abdominal pain and mass, which could be classified in the differential diagnoses of other gastrointestinal disorders. In our case, TLS development accompanied by hypercalcemia alongside high LDH levels in plasma made a differential diagnosis of colorectal cancer less probable and guided us toward hematologic malignancies. Notably, although hypercalcemia is not a routine finding in TLS, hematologic malignancies could raise serum level of calcium despite of TLS occurrence, which should not misguide physicians from diagnosing TLS in a certain clinical setting. Since immediate and intensive action and treatment is required in patients with a life‐threatening oncologic emergency (in this case BL and TLS), a high degree of clinical suspicion is needed to make the correct early diagnosis and proceed with appropriate management and treatment to prevent further complications.

## AUTHOR CONTRIBUTIONS

MS contributed to developing the research idea, composing, and revising the manuscript. ES contributed to developing the research idea, composing, and revising the manuscript. HG contributed to composing and revising the manuscript. NZ contributed to developing the research idea and revising the manuscript. SP contributed to developing the research idea and revising the manuscript.

## CONFLICT OF INTEREST STATEMENT

The authors have no conflict of interest to declare.

## ETHICAL APPROVAL

This study was approved by the research and ethics committee of Tehran University of Medical Sciences. The patient has given her informed consent to publish this case.

## CONSENT

Written informed consent was obtained from the patient for publication of this case report and any accompanying images. A copy of the written consent is available for review by the Editor‐in‐Chief of this journal.

## Data Availability

Data sharing is not applicable to this article as no datasets were generated or analyzed during the current study.
